# Combined vemurafenib and fotemustine in patients with BRAF V600 melanoma progressing on vemurafenib

**DOI:** 10.18632/oncotarget.10589

**Published:** 2016-07-13

**Authors:** Paola Queirolo, Francesco Spagnolo, Virginia Picasso, Laura Spano, Enrica Tanda, Valeria Fontana, Laura Giorello, Domenico Franco Merlo, Ester Simeone, Antonio Maria Grimaldi, Marcello Curvietto, Michele Del Vecchio, Paolo Bruzzi, Paolo Antonio Ascierto

**Affiliations:** ^1^ Department of Medical Oncology, IRCCS AOU San Martino, IST Istituto Nazionale per la Ricerca sul Cancro, Genova, Italy; ^2^ Department of Plastic and Reconstructive Surgery, IRCCS AOU San Martino, IST Istituto Nazionale per la Ricerca sul Cancro, Genova, Italy; ^3^ Department of Epidemiology, Biostatistics and Clinical Trials, IRCCS AOU San Martino, IST Istituto Nazionale per la Ricerca sul Cancro, Genova, Italy; ^4^ Melanoma, Cancer Immunotherapy and Innovative Therapy Unit, Istituto Nazionale Tumori Fondazione “G. Pascale”, Napoli, Italy; ^5^ Department of Medical Oncology, Fondazione Istituto di Ricovero e Cura a Carattere Scientifico (IRCCS) Istituto Nazionale dei Tumori, Milan, Italy

**Keywords:** vemurafenib, BRAF, fotemustine, melanoma, treatment beyond progression

## Abstract

**Background:**

BRAF inhibitor vemurafenib achieves high response rate and an improvement in survival in patients with BRAF-mutated metastatic melanoma. However, median progression-free survival is only 6.9 months in the phase 3 study. Retrospective analyses suggest that treatment with BRAF inhibitors beyond initial progression might be associated with improved overall survival. We aimed to prospectively investigate the activity of prolonged treatment with vemurafenib and the addition of fotemustine in patients with systemic progression on prior single-agent BRAF inhibitor.

**Patients and Methods:**

In this two-centres, single-arm Phase 2 trial, we enrolled patients with systemic progressive disease during single-agent vemurafenib treatment. Participants received vemurafenib 960 mg twice daily or dose administered at time of disease progression with vemurafenib previous treatment and fotemustine 100 mg/m2 intravenously every three weeks. The primary endpoint was PFS.

**Results:**

Thirty-one patients were enrolled in the study; 16 patients had brain metastases at baseline. Median PFS was 3.9 months and 19 patients (61.3%) achieved disease control (1 CR, 4 PR, 14 SD). For patients achieving disease control, median duration of treatment was 6 months. Median OS was 5.8 months from enrolment and 15.4 months from start of previous vemurafenib. Five patients (16.1%) had a G3-4 AE, the most common being thrombocytopenia, which occurred in 3 patients.

This trial is registered with ClinicalTrials.gov number NCT01983124.

**Conclusion:**

The combination of vemurafenib plus fotemustine has clinical activity and an acceptable safety profile in BRAF-refractory patients.

## BACKGROUND

The identification of BRAF V600 somatic mutations in melanoma [1] led to the development of molecularly targeted therapies, which improved the prognosis of metastatic melanoma patients compared to chemotherapy [2-6]. BRAF inhibitors vemurafenib and dabrafenib achieved improved overall survival (OS) and/or progression free survival (PFS) over chemotherapy and have been approved for the treatment of BRAF-mutated metastatic melanoma. More recently, combined BRAF and MEK inhibition out-performed BRAF inhibitors monotherapy in three randomized Phase 3 trials [5-9] and the combinations of dabrafenib plus trametinib and vemurafenib plus cobimetinib are now approved by the FDA and EMA for the treatment of metastatic melanoma with a BRAF V600E or V600K mutation. However, the majority of patients face progressive disease (PD) even when treated with the combination due to the development of a variety of mechanisms of acquired resistance [10-12]. Patterns of progressions to BRAF inhibitors therapy are heterogeneous [13]. Often, progressive lesions are only a small amount of the total tumour burden, thus a local treatment of such lesions can be provided with ongoing systemic treatment. Among 48 patients enrolled in the Phase I study of vemurafenib, 20 patients continued vemurafenib > 30 days after local therapy of PD lesions; among these 20 patients, median OS was 26.0 months from initiation of vemurafenib and 10.0 months beyond initial PD, with a median treatment duration beyond progression of 3.8 months. Conversely, in patients who did not continue vemurafenib after progression, median OS was 11.0 from initiation of vemurafenib and 3.4 months after PD [14]. Similarly, treatment beyond progression was associated with better OS in a retrospective analysis conducted by Chan and colleagues [15] on a series of patients treated with BRAF inhibitors vemurafenib and dabrafenib as single agents in phase I/II/III/IV trials and in another retrospective analysis by Scholtens and colleagues [16].

Even if a local treatment cannot be delivered, some evidence support continuation of BRAF inhibitor treatment, as partial suppression of the reactivated MAPK pathway can occur [15, 17]. Molecularly targeted drugs have demonstrated clinical benefit when used beyond disease progression in other solid tumors [18-19]; however, currently, no prospective data exist supporting extended BRAF inhibition beyond local or systemic progression. We conducted a Phase II trial to assess the clinical activity of continued BRAF inhibition, with the addition of chemotherapy, in patients with BRAF-mutated metastatic melanoma and systemic PD during vemurafenib treatment as single agent. Before the approval of anti-PD-1 agents, chemotherapy was the standard of care for BRAF-mutated patients after the failure of BRAF inhibitors if pre-treated with ipilimumab or with low life expectancy, as expected for many BRAF-refractory patients with systemic progression. As we predicted a high rate of patients with central nervous system involvement, we chose fotemustine as chemotherapy drug because it has clinical activity in patients with brain metastases [20].

## MATERIALS AND METHODS

### Study design and patients

This two-center, single-arm Phase 2 study was done to assess the clinical activity, safety, tolerability and efficacy of vemurafenib plus fotemustine in patients with BRAF-mutated unresectable stage IIIC or stage IV melanoma with systemic progressive disease during single-agent vemurafenib treatment. To be eligible for enrolment in the study, patients had to have histologically confirmed unresectable stage IIIC or stage IV metastatic melanoma with a documented BRAF V600 mutation and had to be in systemic progression (i.e. progressive lesions could not be treated, by the judgment of the investigators, with a local or loco-regional treatment) during treatment with single agent vemurafenib according to RECIST1.1 criteria.

The study protocol was approved by the institutional review boards or independent ethics committee at each participating study centre, and the study was done in accordance with the provisions of the Declaration of Helsinki and Good Clinical Practice guidelines. All patients provided written informed consent.

This trial is registered with EudraCT, number 2012-004172-18, and with ClinicalTrials.gov, number NCT01983124.

### Procedures

Screening and on-study tumour assessment and safety procedures included brain/chest/abdomen/pelvis CT or MRI (plus bone scan when clinically indicated); dermatologic and head and neck examination for SCC; haematology and biochemistry; 12-lead ECG.

Following a screening period of maximum 28 days, participants received vemurafenib 960 mg twice daily or dose administered at time of disease progression with vemurafenib previous treatment and fotemustine 100 mg/m2 intravenously every three weeks until progression of disease, unacceptable toxicity, withdrawal of consent, or death. Patients were allowed to continue vemurafenib as single agent during the screening phase. Toxic effects were graded according to the Common Terminology Criteria for Adverse Events (CTCAE), version 4.0. Vemurafenib dose interruption and reduction to 720 mg twice a day and then 480 mg twice a day was allowed for intolerable grade 2 or 3 toxicity. Fotemustine dose modifications, interruptions and delays were allowed according to haematological status and specified in the protocol. Tumour assessments were obtained at baseline, week 6, 12, 18, as per institution standard of care thereafter (but at a minimum every 12 weeks) and at the end of study. Tumor response was assessed with the use of RECIST 1.1 criteria.

### Outcomes

The primary objective was to assess the activity of vemurafenib plus fotemustine in BRAF-mutated patients that recurred while on treatment with vemurafenib. The primary endpoint was PFS. The secondary endpoints were incidence of G3-4 toxicities (any type); to estimate rate, duration of response and proportion of patients with duration of response lasting > 24 weeks; to evaluate disease control rate (proportion with best response of CR+PR+SD); to evaluate time to response; to evaluate the incidence of BM in patients free from BMs at the time of enrolment; overall survival.

### Statistical analysis

The following assumptions were made in the estimation of a required sample size of 30 patients. Based on the phase I study of vemurafenib [21], at the time of study conception the expected median PFS in metastatic melanoma patients progressing while on vemurafenib and treated with chemotherapy alone was less than 2 months. Based on the toxicity profile of vemurafenib, its further use could be justified if associated with a non-negligible increase that is >= 2 months increase (from 6-8 weeks to 12-16 weeks) in PFS, corresponding to a HR of 0.5. This effect was plausible, since in the group continuing vemurafenib median PFS could be estimated as of 3-4 months [14]. Based on these assumptions, the association vemurafenib with fotemustine was considered worth further studies if a median PFS >= 12 weeks was observed. Assuming a median PFS of 8 weeks with chemotherapy alone, the null hypothesis of no effect of vemurafenib could be rejected at the 0.1 (1-sided) level of significance with power=80% if vemurafenib is associated with a HR of 0.5.

Progression free survival (PFS) and overall survival (OS) were estimated using the Kaplan-Meier survival function. Time to progression or to death was computed in months as the difference between the date of progression or of death and the date of administration of vemurafenib and fotemustine. Survival plots, median survival times and the interquartile range were computed using the software IBM SPSS Statistics Version 20.0 (IBM Corp. Released 2012. IBM SPSS Statistics for Windows, Version 20.0. Armonk, NY: IBM Corp.).

## RESULTS

### Patients characteristics and treatment

From January to October 2013 thirty-one patients were enrolled in the study. All screened patients were eligible for enrolment. Patients characteristics at baseline are summarized in Table 1. Sixteen patients (51.6%) had brain metastases at baseline; five patients received WBRT (n=3) or SRS (n=2) before treatment with vemurafenib as single agent and three patients received WBRT (n=2) or SRS (n=1) after PD with vemurafenib. There were no cases of brain progression as only site of disease progression among patients included in the study. Fourteen patients (45.2%) had elevated LDH at baseline and 4 (12.9%) had PS>0. Seven patients (22.6%) received ipilimumab prior to vemurafenib.

**Table 1:** Patients characteristics at baseline

	Study Population (n=31)
**Sex**	
Male	14 (45.2%)
Female	17 (54.8%)
**Age**	
Median (IQR)	45.5 (22.6%)
Mean (SD)	51.8 (14.4%)
**Metastatic melanoma stage**	
M1a	4 (12.9%)
M1b	5 (16.1%)
M1c	22 (71.0%)
**Brain metastases**	
Yes	16 (51.6%)
No	15 (48.4%)
**Lactate dehydrogenase concentration**	
Normal	17 (54.8%)
Increased	14 (45.2%)
Missing	0
**ECOG PS**	
0	27 (87.1%)
1	4 (12.9%)
≥2	0
**Previous systemic treatment for metastatic disease** (other than vemurafenib)	
Chemotherapy	10 (32.3%)
Ipilimumab	7 (22.6%)
Both	7 (22.6%)
**Number of previous systemic treatment for metastatic disease** (other than vemurafenib)	
0	19 (61.3%)
1	1 (22.6%)
2	3 (9.7%)
>2	2 (6.4%)

**Figure 1:**
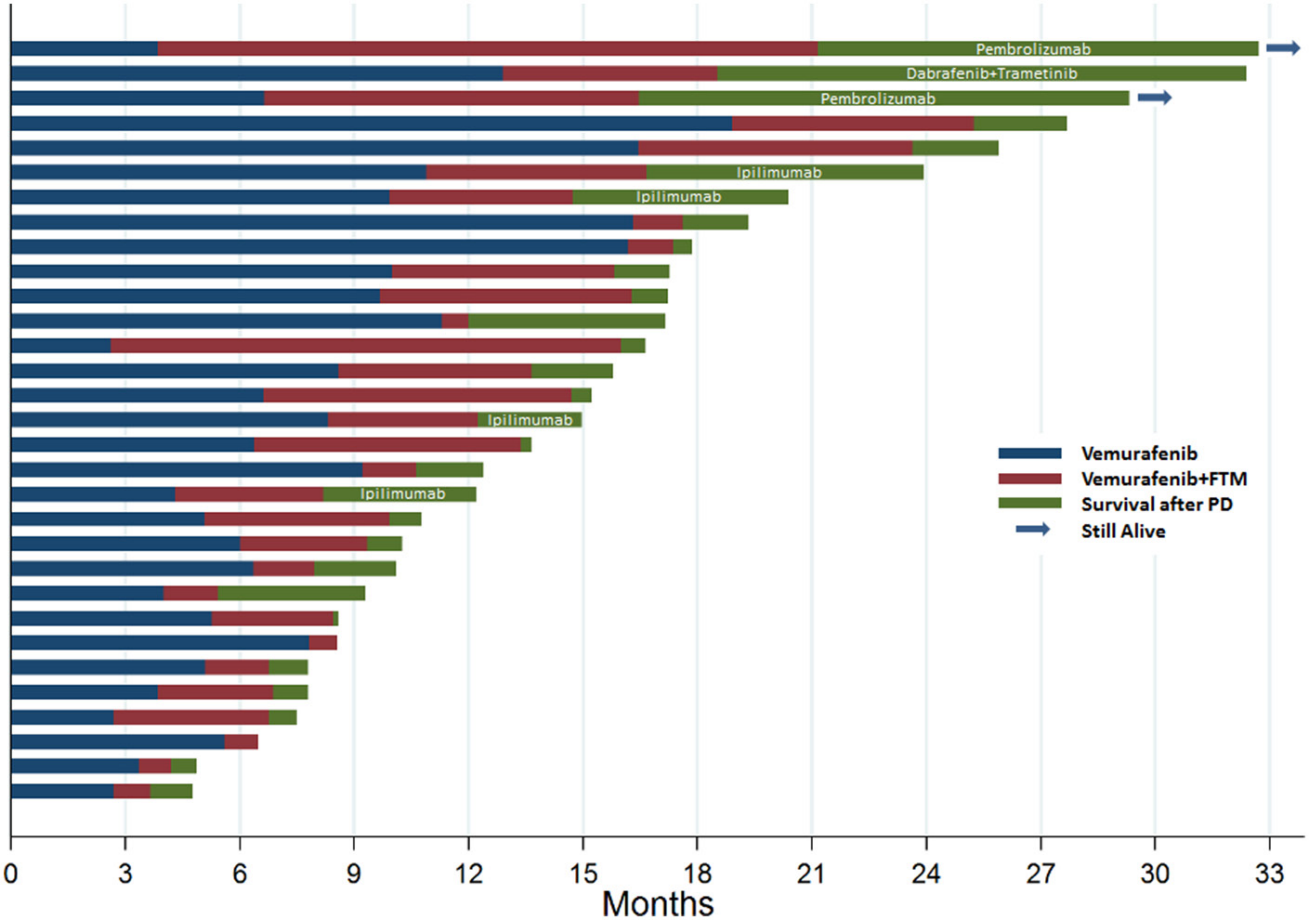
The swimmers plot illustrates the survival of patients treated with vemurafenib as single agent prior to enrolment (blue), with vemurafenib and fotemustine within the study (red) and after progressive disease (green) The screening period (maximum of 28 days) prior to enrolment in the study is not displayed.

Median PFS on prior vemurafenib as single agent was 6.6 months (IQR 4.7-9.9) (Figure 1).

### Clinical activity and efficacy

**Figure 2:**
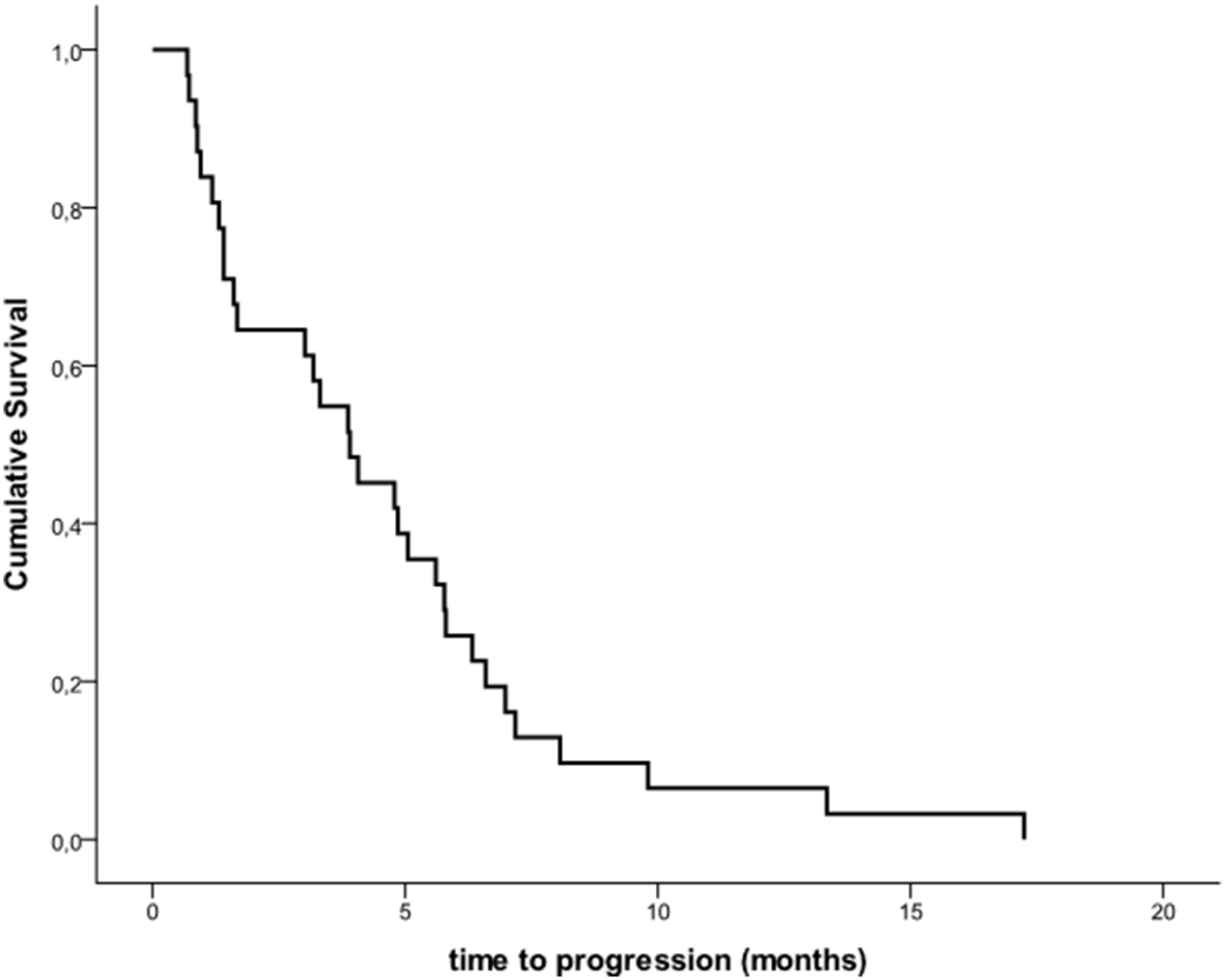
Kaplan-Meier Estimates of Progression-free Survival

Median PFS was 3.9 months (IQR 1.4-6.3) (Figures 1-2). Overall response rate was 16.1%, including 1 CR and 4 PRs; of these, 3 responses were confirmed in at least a subsequent tumor assessment. Fourteen patients achieved SD, for an overall DCR of 61.3%. For patients achieving disease control, median duration of treatment was 6 months. Three patients (9.7%) had a response lasting >24 weeks.

In the 16 patients with brain metastases at baseline, 1 patient (6.3%) had an intracranial PR and 10 patients (62.5%) had SD of brain metastases as best response, for an intracranial disease control rate of 68.8%. All 16 patients eventually had PD: 5 patients (31.3%) had PD in extra-CNS sites only, whilst 11 patients (68.8%) had PD of brain metastases (4 patients in the CNS only and 7 patients in both visceral and CNS localizations), with a median time to progression of brain metastases of 3 months. Incidence of brain metastases in patients free from CNS involvement at the time of enrolment was 13.3%.

**Figure 3:**
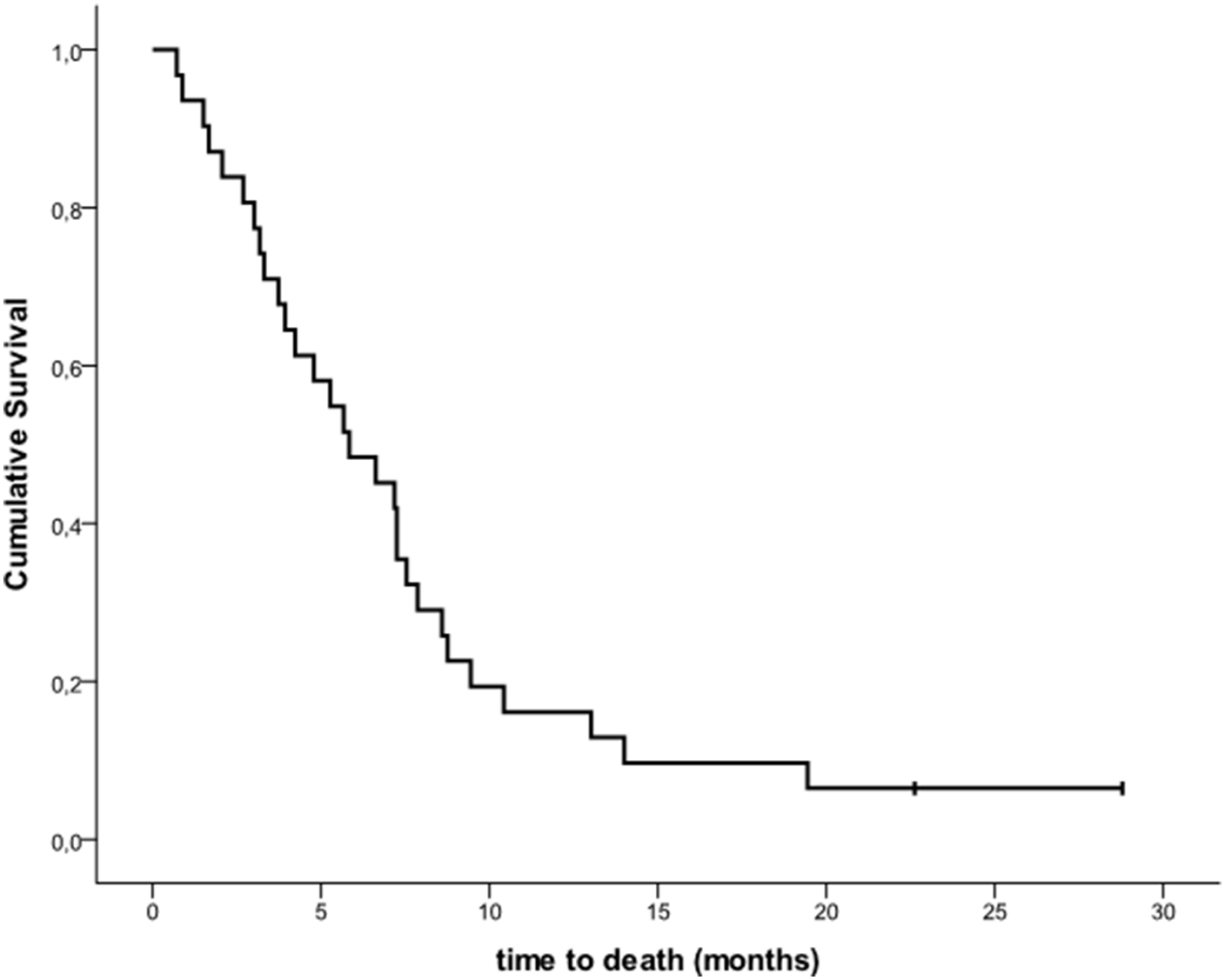
Kaplan-Meier estimates of overall survival

Median OS was 5.8 months (IQR 3.1-8.7) (Figure 3) from start date of vemurafenib+fotemustine and 15.4 months from start date of previous vemurafenib treatment as single agent. Median OS for patients with brain metastases at baseline was 5.8 months (*vs.* 5.7 months for patients without brain metastases) from start date of vemurafenib+fotemustine and 14.0 months (*vs.* 18.8 for patients without brain metastases) from start date of previous vemurafenib treatment as single agent.

During the follow-up period, 4 patients received ipilimumab, 1 patient dabrafenib+trametinib and 2 patients received an anti-PD-1 agent after they had disease progression while receiving vemurafenib plus fotemustine (Figure 1).

### Safety

Twenty-six (83.9%) patients had an adverse event (AE) of any grade; 5 patients (16.1%) had a G3 AE. No grade 4 AEs were reported. The most common G3 AE was thrombocytopenia, which occurred in 3 patients. AEs reported by at least 5% of patients are summarized in Table 2. Eight patients (25.8%) entered the study at a reduced dose of vemurafenib: 7 patients (22.6%) at 720 mg twice daily and 1 patient (3.2%) at 480 mg twice daily. After study entry, vemurafenib dose reduction was necessary for 3 patients (9.7%); fotemustine dose delays occurred in 14 patients (45.2%) and dose reduction in 7 patients (22.6%), mostly due to thrombocytopenia.

**Table 2:** Adverse events reported by at least 5% of patients irrespective of association with treatment

	Any Grade	Grade 2	Grade 3
Anemia	11	4	0
Leucopenia	7	0	0
Neutropenia	2	0	0
Thrombocytopenia	11	6	3
Increased ALT	9	3	0
Increased AST	3	0	0
Increased ALP	2	0	0
Increased GGT	11	4	1
Increased Creatinine	3	0	0
Increased Blood bilirubin	5	2	1
Diarrhea	2	1	0
Nausea	3	0	0
Edema limbs	2	0	0
Fatigue	11	4	1
Anorexia	3	0	0
Weight loss	2	1	0
Cutaneous rash	3	1	0
Headache	2	1	0
Myalgia	3	3	0
Pain	3	2	0
Cough	2	0	0

## DISCUSSION

The prognostic characteristics of our study population were highly unfavourable, with more than half patients having brain metastases and/or elevated LDH levels at baseline, and were more similar to a “real world” experience than a clinical trial with selected patients. In addition to that, all patients were facing systemic progressive disease during treatment with BRAF inhibitors.

In the safety study of vemurafenib [6], patients were more representative of routine clinical practice compared to registration clinical trials and their characteristics were more similar to those of our study population, with 50% of patients having elevated LDH levels (*vs*. 45%) and 23% having brain metastases (*vs*. 52%) at baseline. Median OS for the overall population was 12.0 months; in patients with and without brain metastases median OS was 6.2 and 15.2 months, respectively. Notably, in our study, median OS from start date of vemurafenib as single agent in the overall population was similar to median OS observed in patients without brain metastases of the vemurafenib safety study (15.4 *vs*. 15.2 months); moreover, in our study, median OS from start date of vemurafenib+fotemustine in patients with brain metastases was similar to median OS observed in patients with brain metastases of the vemurafenib safety study (5.8 *vs.* 6.2 months). These results, despite the limitations due to the small sample size of our study, suggest that vemurafenib treatment beyond progression and fotemustine may be particularly effective in patients with brain metastases. Remarkably, in our study, median OS from start date of vemurafenib+fotemustine was similar for patients with and without brain metastases at baseline (5.8 *vs.* 5.7 months). It should be acknowledged that, as many patients with brain metastases were included, disease evaluation with brain MRI in all patients would have been more accurate, whilst most patients included in our study were evaluated with CT-scan.

No prospective data from randomized trials exist comparing different treatment strategies after progression with BRAF inhibitors, but some data suggest that ipilimumab and MEK inhibitors either as single agents or in combination with BRAF inhibitors may have limited efficacy in BRAF-refractory patients. Though available data are retrospective and heavily biased, patients treated with ipilimumab after BRAF inhibitors seem to have poor survival outcomes [22-24], with almost half patients having rapid PD and dying before completing all four doses [24]. No objective responses were observed in two studies where MEK inhibitors as single agents were given after progression with BRAF inhibitors [25-26].

The addition of a MEK inhibitor to a BRAF inhibitor regimen at progression has modest clinical activity: in the phase I/II study of dabrafenib and trametinib, median PFS was only 3.6 months with an overall response rate as low as 13-15% in 71 patients treated with the combination at progression with dabrafenib monotherapy [27] and in the Phase 1b study of vemurafenib plus cobimetinib (BRIM-7), only ten (15%) of 66 patients who had progressed on vemurafenib had an objective response, with a PFS of 2.8 months [28]. Median OS from the start of combination therapy was 10-11.8 months in the dabrafenib plus trametinib and 8.3 months in BRIM-7. The setting of these two studies, i.e. patients with progressive disease on single agent BRAF inhibitor, was similar to that of our current study, even though less patients had brain metastases at baseline (14% in the dabrafenib plus trametinib study *vs*. 52% in our study; not reported in BRIM-7).

Pre-clinical *in vitro* data showed that combined treatment with vemurafenib plus fotemustine has an additive effect on cell kill and that acquired resistance to BRAF inhibition does not influence the activity of fotemustine [29]. Chemotherapy may be of benefit in heterogeneous tumours, where some cells are still sensitive to BRAF inhibition whilst the others acquired resistance. Nevertheless, we do not know whether fotemustine contributed to the efficacy observed in our study or if the benefit primarily derived from treatment beyond progression with BRAF inhibitors.

This is the first study assessing the safety and clinical activity of vemurafenib in combination with chemotherapy. This novel combination regimen was safe and no unexpected adverse events were observed. Patients were already on vemurafenib treatment at the time of enrolment and in some of them vemurafenib dose was already reduced, so a low rate of adverse events attributable to vemurafenib was reported, and fotemustine toxicity was easily manageable with dose delay/modifications.

## CONCLUSION

Current options for patients after PD with BRAF+/-MEK inhibitors include ipilimumab and anti-PD-1 agents; anti-PD-1 drugs are effective regardless BRAF mutational status [30] and after treatment with BRAF inhibitors in BRAF-mutated patients [31]. At the time of the conduction of our study, these options were limited to ipilimumab or chemotherapy. The combination of vemurafenib plus fotemustine has clinical activity and an acceptable safety profile in patients with systemic progression on prior single-agent BRAF inhibitor. BRAF inhibitors as single agents are not the standard treatment for BRAF-mutated patients anymore, since the combination of BRAF and MEK inhibitors outperformed monotherapy in three phase 3 clinical trials [12], and the combination of vemurafenib plus fotemustine should not be studied any further; however, we believe that further investigation is warranted into the impact of MAP-kinase inhibitors treatment beyond progression on survival. For this reason, we planned a randomized phase 2 study to evaluate the efficacy beyond progression of vemurafenib in combination with cobimetinib compared to an investigator’s choice second line treatment in BRAF-mutated patients refractory to a first line therapy with vemurafenib and cobimetinib.
